# Hybrid MLOps framework for automated lifecycle management of adaptive phishing detection models

**DOI:** 10.1038/s41598-025-23600-z

**Published:** 2025-11-04

**Authors:** Asmaa Reda, Shereen A. Taie, Masoud E. Shaheen

**Affiliations:** Computer Science Department, Faculty of Computers and Artificial Intelligence, Fayoum University, Fayoum, 63514 Egypt

**Keywords:** Adaptive machine learning, MLOps framework, Phishing detection, Concept drift, Automated model retraining, Responsible AI, Model robustness, Automated drift detection, Cybersecurity operations, Engineering, Mathematics and computing

## Abstract

Phishing detection models degrade quickly due to drift, adversarial evasion, and fairness issues. Existing MLOps platforms mainly automate deployment and monitoring. Prior works have examined SHAP-based monitoring, retraining, or fairness audits separately, but lack an integrated theory of resilience for adversarial environments. We introduce the Hybrid MLOps Framework (HAMF), a system designed to embed resilience and ethical governance into the lifecycle of phishing detection models. HAMF is ‘hybrid’ because it unifies proactive and reactive adaptation, combining automation with stakeholder oversight, and embedding resilience with ethical governance. HAMF treats resilience as an integrated lifecycle property, designed to simultaneously preserve model accuracy, fairness, and stakeholder trust amidst concept drift. Methodologically, HAMF implements this through a hybrid control cycle. This cycle fuses four key capabilities: SHAP-guided feature replacement, event-driven retraining, fairness-triggered audits, and structured human feedback. Unlike conventional pipelines where these functions are isolated, HAMF ensures their interdependence as first-class triggers. Empirical evaluations on large-scale phishing streams demonstrate HAMF’s superior performance. The framework detects drift within 18 seconds, restores F1 scores above 0.99 post-attack, reduces subgroup disparities by over 60%, and scales to over 2,300 requests per second with sub-50ms latency. These results validate HAMF’s design, demonstrating that embedding resilience and ethical alignment into the MLOps lifecycle is both effective and scalable.

## Introduction

Phishing detection is a persistent challenge. Phishing detection is a persistent and escalating challenge. The Anti-Phishing Working Group (APWG) observed 1,130,393 phishing attacks in the second quarter of 2025, a steady increase from the previous quarter, underscoring the relentless growth of this threat^1^. Attackers constantly change lexical, structural, and behavioral patterns, causing models to degrade and false alarms to rise^2–4^. This deterioration is driven by two main factors: concept drift, where data distributions shift^5–8^ —and feature obsolescence, as commonly used signals such as domain rankings or WHOIS attributes become unreliable or deprecated^9–11^. Bias in phishing detection systems often harms small or low-traffic entities, reducing trust in automation^12–15^.

To address lifecycle management at scale, a range of Machine Learning Operations (MLOps) frameworks have been developed, including Kubeflow^16^, MLflow^17^, TensorFlow Extended (TFX)^18^, and Amazon SageMaker^19^. These platforms provide infrastructure for deployment, reproducibility, and continuous integration/deployment (CI/CD). However, they are limited in four respects. First, they are designed for stationary environments and lack robust mechanisms for detecting real-time model degradation. Second, they provide no systematic support for dynamic feature substitution when upstream signals fail. Third, fairness and ethical compliance are externalized rather than embedded lifecycle properties^12,14,20^. Finally, human feedback is generally ad hoc, rather than operationalized as a structured component of retraining workflows^12^. As a result, current MLOps pipelines remain vulnerable in adversarial and high-stakes domains such as phishing detection.

This study introduces HAMF – the Hybrid MLOps Framework, which advances beyond existing systems in both concept and methodology. The term hybrid reflects its integration of proactive and reactive adaptation, combining automated retraining with stakeholder oversight, and aligning technical resilience with fairness-aware governance. Conceptually, HAMF redefines resilience as a closed-loop lifecycle property that simultaneously maintains accuracy, fairness, and trust continuity under drift. Methodologically, it introduces a hybrid control cycle that fuses SHAP-guided feature replacement^3,21^, event-driven retraining^10^, fairness-triggered auditing^12,14,15^, and stakeholder-in-the-loop feedback into a unified pipeline. Unlike existing platforms, where these elements are isolated or optional, HAMF enforces their interdependence as first-class lifecycle triggers.

This work makes the following contributions:

- **Conceptual contribution** – Introduces the notion of resilience-by-design in adversarial MLOps, defined as the joint preservation of accuracy, fairness, and trust continuity under concept drift and feature volatility.
- **Methodological contribution** – Proposes a hybrid closed-loop control cycle that integrates SHAP-guided feature replacement, event-driven retraining, fairness-aware auditing, and stakeholder-in-the-loop oversight as interdependent lifecycle triggers.
- **Architectural contribution** – Provides a modular, microservices-based framework that embeds explainability, fairness, and compliance as first-class operational properties, surpassing general-purpose MLOps systems such as Kubeflow, MLflow, TFX, and SageMaker^17–19^.

The remainder of the paper is organized as follows. Section 2 reviews related work. Section 3 presents the HAMF architecture, followed by the implementation methodology in Sect. 4. Section 5 reports experimental results, including ablation studies and benchmarking. Section 6 discusses ethical, legal, and interpretability considerations. Section 7 outlines the implications and limitations. Section 8 concludes with directions for future work.

## Related work

This section reviews the research landscape that informs the motivation and design of HAMF. We structure the discussion into three thematic areas:(1) Core MLOps Infrastructure, which covers foundational platforms and automation;(2) Adaptation and Monitoring, focusing on handling dynamic environments through drift detection and ethical auditing; and(3) Domain-Specific Applications, highlighting the unique challenges in phishing detection. A comparative synthesis concludes the section, summarizing key gaps and HAMF’s advancements.

### Core MLOps infrastructure and automation

#### MLOps frameworks

MLOps platforms such as Kubeflow^16^, MLflow^17^, TensorFlow Extended (TFX)^18^, and Amazon SageMaker^19^ have established standards for deployment automation, artifact tracking, and CI/CD integration. They support reproducibility and scalability but lack real-time drift handling and fairness auditing. Recent efforts have explored integrating fairness monitoring into pipelines^14^, and novel architectures for automation in DevOps^15^. However, they lack comprehensive lifecycle governance, especially for adversarial conditions.

#### Automated model maintenance

Automated maintenance often uses scheduled retraining or manual fixes, which fail in dynamic phishing settings. Earlier works emphasized continuous integration and DevOps-style orchestration^12^, while more recent methods focus on event-driven retraining and drift-aware updating^10^. End-to-end automation pipelines^19^ and business process drift monitoring^22^ have demonstrated progress, but they generally lack mechanisms for feature substitution or stakeholder-guided interventions, both critical in adversarial cybersecurity.

### Adaptation and monitoring

#### Concept drift and feature volatility

Concept drift adaptation has been extensively studied through methods such as ADWIN^6^, the Early Drift Detection Method (EDDM)^5^, and adaptive windowing^7^. More recent research emphasizes interpretability-driven explanations of drift^23^ and systematic reviews of text stream adaptation^9^.While these methods effectively detect drift, they often overlook feature volatility—the obsolescence or adversarial manipulation of features^10,24^. HAMF advances this field by combining drift detection with SHAP-based feature replacement, ensuring resilience against both distributional shifts and semantic feature failures.

####  Fairness auditing and bias mitigation

Fairness-aware machine learning has become a central concern, with toolkits such as AI Fairness 360^25^ enabling detection and mitigation of algorithmic bias. Surveys^13,15^ highlight the persistent gaps in fairness integration into operational pipelines, while recent works argue for fairness-aware engineering practices^14^. Ethical auditing frameworks such as the NIST AI Risk Management Framework^26^ and discussions on ethics-by-design^20,27^ stress the importance of embedding governance directly into ML workflows. Explainability techniques such as SHAP^3,21^ are widely used, but their integration as active triggers for fairness-aware retraining is a novel contribution of our work. HAMF operationalizes these concerns by treating fairness and transparency as first-class lifecycle triggers, not post hoc analyses.

### Domain-specific applications: phishing detection systems

Phishing detection has traditionally relied on lexical, structural, and behavioral feature analysis^2,3^, with frameworks such as PhishHaven^4^ and PhishBench 2.0^28,29^ providing domain-specific benchmarking. Recent advancements leverage ensemble learning^3^, adversarial evaluation^30^, and adaptive detection strategies. However, existing phishing detection systems rarely integrate automated drift handling, fairness auditing, or human feedback mechanisms. The growing concern of AI-driven attacks^20,26^ underscores the urgency of developing pipelines that not only detect phishing but also maintain fairness and resilience in adversarial contexts.

### Comparative summary

As summarized in Table 1, conventional MLOps platforms prioritize reproducibility and deployment scalability but lack core capabilities such as feature-level resilience, fairness-aware retraining, and contextual stakeholder collaboration. HAMF unifies these features into a domain-specific framework optimized for adversarial, high-risk settings such as phishing detection.

**Table 1:** Comparative Analysis of HAMF and Prominent MLOps Frameworks.

Capability	Proposed HAMF	Kubeflow ^16^	MLflow ^17^	TFX ^18^	Amazon SageMaker^19^
End-to-End Lifecycle Management	✓	✓	$\blacktriangle$ $\phantom{0}^{\ddagger }$	✓	✓
Automated Event-Driven Retraining	✓	✗	✗	✗	$\blacktriangle$ $\phantom{0}^{\P }$
Dynamic Feature Replacement	✓	✗	✗	$\blacktriangle$ $\phantom{0}^{\S }$	✗
Fairness & Ethical Auditing	✓	✗	✗	✗	$\blacktriangle$ $\phantom{0}^{\P }$
Explainability (Integrated SHAP)	✓	$\blacktriangle$ $\phantom{0}^{\dagger 2}$	$\blacktriangle ^{\ddagger }$	$\blacktriangle$ $\phantom{0}^{\S }$	✗
Drift Detection (Real-Time)	✓	$\blacktriangle ^{\dagger 1}$	✗	$\blacktriangle ^{\S }$	$\blacktriangle ^{\P }$
Stakeholder-in-the-loop Feedback	✓	✗	✗	✗	✗
Cloud Independence / Portability	✓	$\blacktriangle$ $\phantom{0}^{\S }$	✓	$\blacktriangle$ $\phantom{0}^{\S }$	✗
Scalability (Distributed / Parallel)	✓	✓	$\blacktriangle$ $\phantom{0}^{\ddagger }$	✓	✓
Open-Source & Extensibility	✓	✓	✓	✓	✗

✓ Supported    ✗ Not Supported    $\blacktriangle$ Partially Supported or Requires Manual Setup

$\phantom{0}^{\dagger 1}$ Requires custom implementation of drift detection algorithms (e.g., PSI, KL-divergence) as pipeline components, as it is not a native feature^16^.

$\phantom{0}^{\dagger 2}$ Supports explainability only if manually integrated with external tools like SHAP or Lime; not provided out-of-the-box^16^.

$\phantom{0}^{\ddagger }$ Provides core tracking but requires integration for full lifecycle management(e.g.,distributed execution relies on external engines like Spark^17^).

$\phantom{0}^{\S }$ The pipeline structure can be manually engineered to support this feature, but it is not provided as a standard, pre-built, or configurable component^18^.

$\phantom{0}^{\P }$ Managed services exist(e.g.,Clarify for fairness), but event-driven retraining requires significant custom workflow configuration^19^

## HAMF framework

The Hybrid Approach MLOps Framework (HAMF) introduces a resilient, modular architecture tailored to adaptive phishing detection. Designed for adversarial, non-stationary domains, HAMF departs from traditional MLOps practices—such as those found in Kubeflow^16^ or SageMaker^31^—by embedding real-time drift handling, explainable feature optimization, and continuous fairness auditing directly into its operational loop.

### Architectural design principles

The Hybrid MLOps Framework (HAMF) is designed to support resilient, adaptive, and ethically aligned model operations in adversarial environments such as phishing detection. Unlike conventional MLOps platforms that prioritize automation and scalability^17,18^ , HAMF embeds resilience-by-design as a first-class property. Here, we define resilience as the ability to sustain predictive accuracy, fairness, and stakeholder trust despite concept drift, feature volatility, and adversarial attacks^5,6,8,9^.

To achieve this, HAMF adheres to three guiding principles:

1. **Closed-loop monitoring and adaptation **—Continuous detection of drift and feature instability, coupled with event-driven retraining and feature replacement^9,10^.
2. **Fairness-aware lifecycle control**—Integration of fairness audits and bias mitigation as triggers for retraining and deployment gating, ensuring ethical accountability in high-stakes domains^12,14,15,20^.
3. ** Hybrid human–machine oversight**—Stakeholder-in-the-loop mechanisms that allow experts to validate automated decisions, contribute domain insights, and oversee bias mitigation^12^.

### The principle of trust continuity

A foundational design principle of HAMF is the operationalization of Trust Continuity.We define Trust Continuity as the sustained stakeholder confidence that an adaptive AI system will consistently meet its performance, fairness, and ethical goals as it adapts to a changing environment. It is not a static property but an emergent quality achieved through a continuous cycle of transparent monitoring, explainable adaptation, and auditable governance. This ensures the system’s behavior remains predictable and aligned with human intent despite its dynamic nature.

As real examples, we are listing three representative use cases from daily enterprise operations as follows:

1. In Phishing Detection (URL Analysis): A HAMF-powered system detects novel URL obfuscation techniques as drift and automatically retrains to block them, while its stakeholder feedback loop allows analysts to correct false positives. This dual capability of automated resilience and human-guided correction provides a clear value proposition for the enterprise: it maximizes threat containment against zero-day attacks and minimizes business disruption by ensuring legitimate partners are not incorrectly blocked, thereby protecting both security and operational continuity.
2. In Cybersecurity (Adaptive Network Security): When faced with a zero-day exploit, HAMF provides Security Operations Center (SOC) analysts with a SHAP-based explanation of the new attack vector while automatically adapting security policies. The value for the SOC is a significant acceleration of the incident response lifecycle; by automating detection and providing immediate, explainable insights, HAMF empowers analysts to validate and act on threats faster and with higher confidence, reducing the mean time to resolution (MTTR).
3. In Digital Transformation (AI in Finance): When an AI model for loan approvals adapts to new economic conditions, HAMF’s embedded Ethical Compliance Module ensures the retrained model does not introduce bias against protected groups. The value for the financial institution is the ability to innovate with AI safely and at scale. HAMF de-risks the adoption of adaptive AI in regulated environments by ensuring that models remain both profitable and provably compliant, thereby unlocking new efficiencies while maintaining regulatory trust.

### Modular microservices architecture

HAMF follows a microservices paradigm, where loosely coupled, containerized modules communicate through APIs to ensure fault isolation, observability, and scalability. This modularity enables HAMF to operate across both streaming and batch workflows, accommodating real-time phishing detection while supporting retrospective audits^10,22^.

The architecture integrates five core subsystems:

- ** Data management layer**—Ingests and normalizes heterogeneous phishing-related data while ensuring schema consistency and versioning^29^.
- **Feature adaptation engine**—Detects feature volatility and substitutes unstable signals using SHAP-guided semantic replacements, thereby mitigating upstream feature obsolescence^3,10,21^.
- **Model lifecycle orchestrator**—Implements closed-loop control by coupling drift detection, retraining triggers, and model versioning within a unified workflow^10,19^.
- **Monitoring and feedback layer**—Tracks predictive performance and system health, and integrates stakeholder feedback into retraining decisions^12^.
- **Ethical compliance module**—Embeds fairness auditing and compliance checks aligned with governance standards such as GDPR and the NIST AI Risk Management Framework^15–26^ .

A sixth supportive layer, Documentation and Knowledge Management, records model lineage, feature changes, and audit outcomes, ensuring traceability and reproducibility^32^.

### Architectural overview

**Figure 1:**
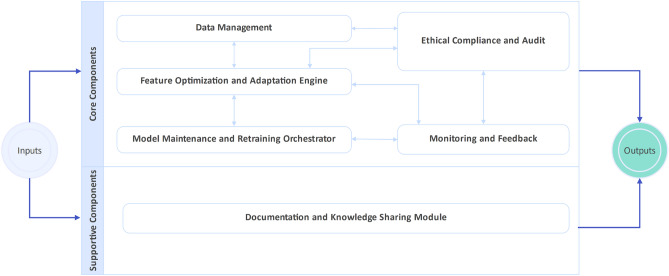
HAMF Modular Architecture.

Figure 1 illustrates HAMF’s modular architecture. Each subsystem is loosely coupled, enabling independent updates and fault isolation. Data flows cyclically across ingestion, feature monitoring, model orchestration, and compliance auditing, ensuring that drift signals and fairness diagnostics directly inform retraining. This integrated design is a key differentiator; existing frameworks typically treat explainability and fairness as external add-ons, whereas HAMF embeds them as core lifecycle triggers^12–15^.

### Distinction from prior frameworks

While existing platforms such as Kubeflow, MLflow, TFX, and SageMaker provide automation for deployment and monitoring^17–19^ , they lack integrated support for feature-level resilience, fairness-aware retraining, and structured stakeholder governance. HAMF advances beyond these systems by:

- Treating explainability, fairness, and resilience not as optional add-ons but as mandatory, interdependent lifecycle properties. This deep integration is empirically shown to reduce fairness disparities to a $\Delta$DP of 0.03, a significant improvement over the baseline frameworks, which exhibited disparities ranging from 0.08 to 0.19 (Table 3).
- Introducing a hybrid control cycle that fuses SHAP-based feature adaptation, event-driven retraining, and fairness-triggered audits into a continuous feedback loop. The operational impact of this hybrid cycle is a dramatic reduction in response time to adversarial threats; our results in Section 5.2 demonstrate that HAMF detects and initiates recovery from adversarial drift in just 18 seconds, whereas comparable platforms require over 90 seconds or rely on simulated manual intervention times of 300 seconds.
- Positioning human oversight as a formal component of the pipeline, ensuring accountability and contextual alignment in security-critical domains.

This combination of architectural modularity and closed-loop resilience operationalizes a new scientific paradigm for adaptive MLOps, distinct from prior frameworks that emphasize scalability without adversarial robustness.

## Methodology

**Figure 2:**
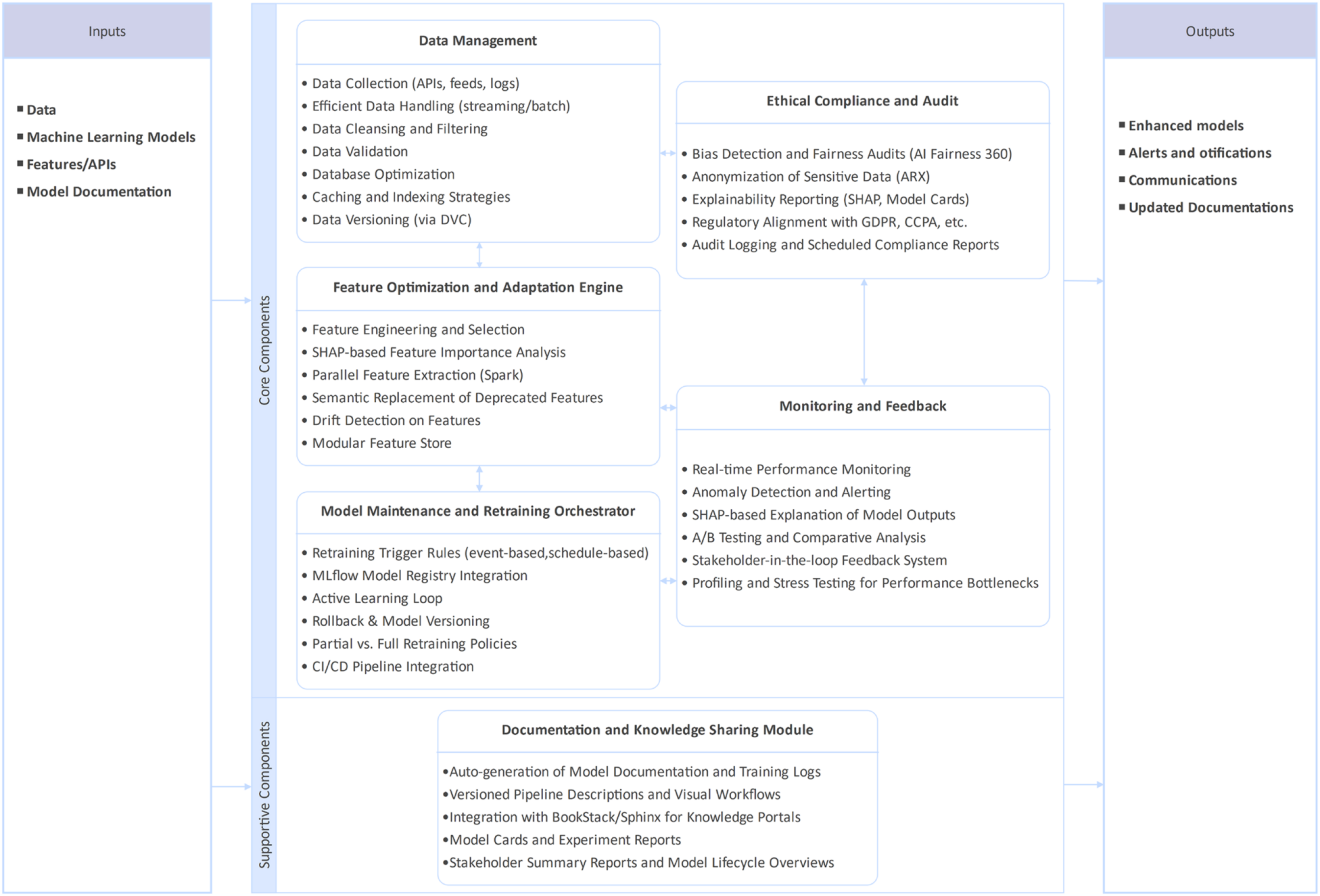
Detailed Component Architecture of the HAMF Framework.

**Figure 3:**
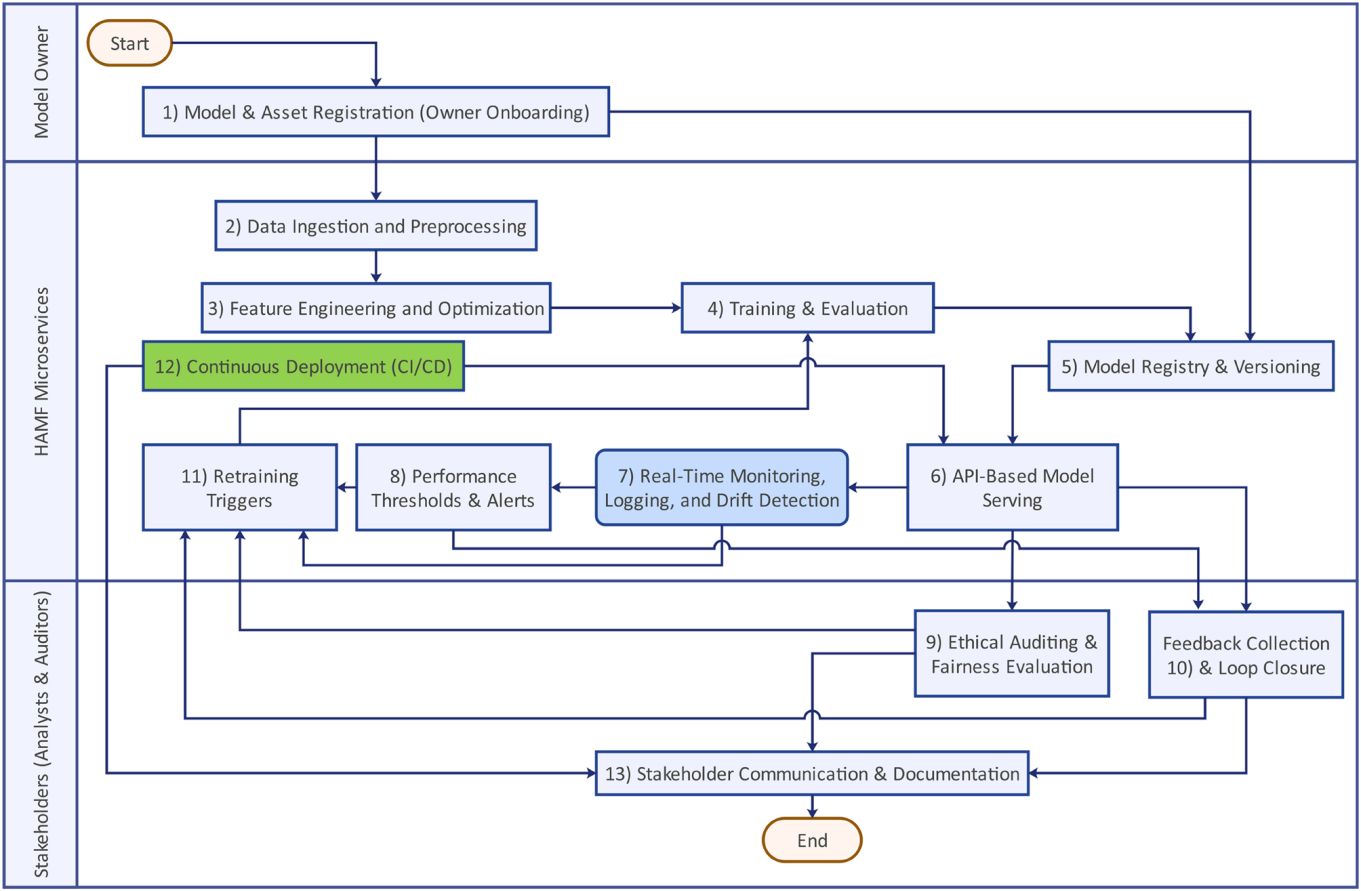
HAMF Workflow.

This section details HAMF’s methodology, translating the architecture from Sect. 3 into a concrete, microservices-based implementation. Figure 2 illustrates this detailed component architecture, mapping each conceptual subsystem to its implemented service and key technologies. This foundation enables a reproducible execution pipeline of thirteen interoperable stages (Figure 3). Each stage is containerized, auditable, and connected via event-driven orchestration. This design ensures continuous adaptation to concept drift, feature volatility, and fairness violations.

### Technology stack and deployment environment

HAMF is implemented in a cloud-agnostic, containerized environment to ensure portability and reproducibility. The technology stack follows a layered design, with each layer selected to balance scalability, explainability, and compliance with ethical standards.

- Data Layer: Distributed processing engines (e.g., Apache Spark) handle both batch and streaming ingestion from phishing intelligence feeds, while relational and object stores ensure ACID-compliant persistence and versioned datasets.
- Model Layer: Training and evaluation are performed using widely adopted ML frameworks (e.g., TensorFlow, XGBoost), with SHAP providing feature attribution to support resilience and interpretability. Lifecycle management is governed by model registries that enforce traceability and semantic versioning.
- Monitoring Layer: System health, drift, and fairness metrics are continuously tracked using a combination of real-time telemetry collectors and visualization dashboards, enabling rapid diagnosis of performance degradation.
- Compliance and Governance Layer: Ethical auditing integrates fairness toolkits (e.g., AIF360) and anonymization utilities, ensuring alignment with GDPR, ISO/IEC 27001, and the NIST AI Risk Management Framework.
- Deployment Environment: Microservices are orchestrated via Kubernetes, allowing elastic scaling of inference endpoints and retraining jobs, while Infrastructure-as-Code principles ensure reproducibility across hybrid or multi-cloud deployments.

This layered design avoids dependency on any single vendor or technology and emphasizes modularity, fault isolation, and auditability—key requirements for high-stakes phishing detection environments.

### Pipeline execution workflow

#### Step 1–model & asset registration (owner onboarding)

- **Inputs**: Model ID/version, dataset URIs, compliance regime.
- **Process**: Models, datasets, and features are versioned via DVC and MLflow. A fairness pre-check (AIF360) runs on a 5% data sample.
- **Outputs**: Versioned baseline artifacts, compliance profile.

#### Step 2–data ingestion and preprocessing

- **Inputs**:Streaming phishing feeds (PhishTank, OpenPhish, Twitter crawlers) and benign sources (Alexa, .gov).
- **Process**: Spark preprocessing: remove duplicates, enforce schema, normalize timestamps, anonymize PII^32,33^.
- **Outputs**: Cleaned, versioned datasets.

#### Step 3–feature engineering and optimization

- **Inputs**:Preprocessed datasets.
- **Process**:Performs lexical and structural transformations. Feature relevance is scored using SHAP^3,21^ to flag unstable features for substitution (e.g., AlexaRank $\longrightarrow$ GoogleRank)^10^.
- **Outputs**: Ranked and stable feature set.

#### Step 4–training and evaluation

- **Inputs**:Engineered feature sets.
- **Process**: Models trained with XGBoost, DNN, and ensembles using stratified cross-validation. Evaluation on temporally stratified splits ensures drift realism.
- **Outputs**: Trained model, SHAP interpretability visualizations, performance metrics (precision, recall, ROC-AUC).

#### Step 5–model registry and versioning

- **Inputs**:Trained model artifacts, evaluation metrics, dataset version IDs, SHAP analyses.
- **Process**:Artifacts stored with lineage links to dataset IDs. Semantic versioning ensures traceability^17,32^.
- **Outputs**: Registered model, lineage logs.

#### Step 6–API-based model serving

- **Inputs**:Registered model.
- **Process**: Served as containerized REST API with autoscaling.
- **Outputs**: Production inference endpoints.

#### Step 7–real-time monitoring, logging, and drift detection

- **Inputs**:Live predictions.
- **Process**: Drift detection via KS, PSI, KL divergence^22,27,34^. SHAP deltas track semantic drift^10,23^.
- **Outputs**: Drift alerts, divergence scores, SHAP attribution shifts.

#### Step 8–performance thresholds and alerts

- **Inputs**:Live model metrics (e.g., F1-score, latency), infrastructure telemetry, historical baselines.
- **Process**:Metrics (F1, latency, FP/FN rates) checked against adaptive SLOs.
- **Outputs**: Breach alerts to stakeholders, retraining triggers.

#### Step 9–ethical auditing and fairness evaluation

- **Inputs**:Labeled validation data, sensitive attribute annotations (e.g., region, device), model predictions, policy definitions.
- **Process**: Fairness metrics (($\Delta$DP), ($\Delta$EO)) computed^12,15^. Privacy validated via k-anonymity and l-diversity^32,33^.
- **Outputs**: Fairness reports, compliance triggers.

#### Step 10–feedback collection and loop closure

- **Inputs**: Model outputs and confidence scores, user annotations, flagged errors, retraining triggers
- **Process**: Analysts annotate false positives/negatives; feedback normalized and reintegrated into training.
- **Outputs**: Enriched validation sets, retraining signals.

#### Step 11–retraining triggers

- **Inputs**: Real-time metrics (F1, AUC), drift indicators, feature status logs, user feedback volume.
- **Process**: A composite trigger initiates retraining based on a combination of drift alerts, fairness violations, or feature deprecations:
  $$\text {Trigger}_{\text {retrain}} = {\left\{ \begin{array}{ll} 1, & \text {if } \Delta _{\text {metric}}> \tau _1 \vee \text {PSI} > \tau _2 \vee F_d \in D \\ 0, & \text {otherwise} \end{array}\right. }$$
  where:
  - $\Delta _{\text {metric}}$ is the performance drop (e.g., F1-score decrease)
  - $\text {PSI}$ is the Population Stability Index for distributional drift
  - $\Delta DP$ is the change in Demographic Parity
  - $F_d$ is a deprecated feature in set *D*
  - $V_{fb}$ is the volume of consistent user feedback (e.g., annotations of false positives/negatives) within a sliding time window
  - $\tau _1, \tau _2, \tau _3, \tau _4$ are the respective thresholds
- **Outputs**: New experiment run, retraining pipeline initiation.

#### Step 12–continuous deployment (CI/CD)

- **Inputs**:Validated model packages, API specifications, deployment descriptors
- **Process**: Retrained models deployed with canary testing and rollback.
- **Outputs**: Updated production model, deployment logs.

#### Step 13–stakeholder communication and documentation

- **Inputs**: CI/CD logs, audit outcomes, model metrics
- **Process**: Slack alerts, Trello tasks, BookStack documentation^17,18^.
- **Outputs**: Notifications, compliance records, audit trails.

**Threshold determination:** The thresholds ($\tau$) are not static but are adaptively calibrated based on a moving baseline of recent system performance. For instance, $\tau _1$ for performance drop is initially set to a 5% relative decrease from the model’s baseline F1-score. This baseline is updated after each successful retraining cycle. This adaptive approach ensures that the triggers remain sensitive to significant degradation without causing excessive retraining due to minor, natural fluctuations.

**Human feedback integration:** Qualitative feedback from analysts (e.g., annotating false positives/negatives in Step 10) is quantitatively integrated via the $V_{fb} > \tau _4$ condition. When a predefined volume ($\tau _4$) of similar feedback events (e.g., 50 annotations of a new phishing pattern) is collected within a 24-hour window, it automatically triggers retraining. This formalizes the “Stakeholder-in-the-loop” mechanism, ensuring that domain expert knowledge directly and rapidly influences model adaptation.

To consolidate the preceding components, Figure 3 and Table 2 illustrate the operational workflow of the HAMF pipeline, spanning all thirteen lifecycle stages from initial model registration to stakeholder communication. Moreover, a detailed breakdown of the computational complexity for each of the thirteen pipeline steps is provided in Supplementary Note 3.

**Table 2:** Summary of HAMF Pipeline Steps.

Step	Title	Purpose	Key tools	Outputs
1	Model & Asset Registration	Register models, datasets, features	MLflow, DVC, ARX	Baseline model trigger, dataset versioning
2	Data Ingestion & Preprocessing	Acquire and clean phishing data	Spark, PostgreSQL, Elasticsearch	Cleaned dataset, monthly snapshot
3	Feature Engineering & Optimization	Transform raw data into predictive features	SHAP, Feature Store	Ranked, transformed feature set
4	Training & Evaluation	Train and validate models using engineered features	TensorFlow, Scikit-learn, MLflow	Trained model, SHAP plots, evaluation metrics
5	Model Registry & Versioning	Track model lineage and deployment readiness	MLflow, MinIO, DVC	Registered model with metadata and versioning
6	Model Serving	Deploy models as RESTful endpoints	FastAPI, BentoML, Kubernetes	Live endpoints with telemetry and API schema
7	Monitoring & Drift Detection	Detect drift and monitor performance metrics	Prometheus, Grafana, Alibi Detect	Drift alerts, SHAP deltas, dashboard logs
8	Performance Thresholds & Alerts	Trigger alerts for performance anomalies	Alertmanager, Grafana, Slack	Real-time alerts, retraining signals
9	Ethical Auditing	Evaluate fairness and compliance indicators	AI Fairness 360, SHAP	Fairness reports, bias flags, audit logs
10	Feedback Loop	Capture expert annotations and issue flags	Slack, Trello, DVC	Curated feedback, retraining candidates
11	Retraining Triggers	Initiate model updates from monitoring or feedback	Alibi Detect, Custom Logic	Triggered retraining workflows
12	Continuous Deployment (CI/CD)	Automate reproducible model rollout	GitLab CI/CD, Terraform, Helm	Updated models in production
13	Stakeholder Communication & Documentation	Notify stakeholders and maintain audit trails	Slack, Trello, BookStack	Logs, notifications, compliance documentation

### Algorithmic summary of execution logic

The HAMF lifecycle is governed by a closed-loop control system (see Supplementary Note 2, Algorithm S1 for pseudocode). This system continuously orchestrates data ingestion, feature adaptation using SHAP-based attribution, and model deployment based on triggers from drift detection, performance monitoring, and fairness auditing. A key component is the SHAP-based feature adapter (see Supplementary Note 2, Algorithm S2), which ensures feature resilience by identifying and substituting unstable features based on attribution shifts.

An analysis of the computational complexity of this integrated system confirms its tractability for high-throughput, real-time environments. The time complexity is dominated by data-dependent operations, scaling linearly with the number of records (n) and features (f), while space complexity remains efficient through bounded history logging. The full derivation of the complexity bounds is provided in Supplementary Note 1.

### Parallel processing & scaling challenges

HAMF addresses scalability by implementing multi-layer parallelism across its pipeline. Rather than redesigning each step, parallelization ensures that ingestion, feature monitoring, retraining, and serving remain tractable under adversarial workloads.

Data ingestion is distributed via Apache Spark, enabling real-time processing of high-frequency phishing feeds. Feature attribution and drift monitoring are parallelized across feature partitions, reducing detection latency from minutes in baseline frameworks to under 20 seconds in HAMF experiments. Model training and retraining are accelerated using distributed GPU clusters managed by Kubernetes autoscaling, completing adaptive retraining within 27 seconds during controlled drift scenarios. Model serving is horizontally scaled, sustaining 2,300 requests/s with <50 ms p99 latency during stress tests.

Scaling challenges include (i) handling burst ingestion from social streams, (ii) GPU saturation during retraining, and (iii) fairness audits introducing batch-processing overhead. HAMF mitigates these via micro-batching with backpressure, autoscaling policies, and asynchronous fairness evaluation.

In summary, HAMF’s layered parallelism ensures low-latency adaptation, high-throughput scalability, and fairness-aware monitoring at scale. Unlike general-purpose MLOps platforms, which offer either lifecycle automation or domain-specific drift detection, HAMF combines both within a unified, production-ready pipeline^4,8,10,12,21,22,28^.

### Justification of tools and configuration

The Hybrid MLOps Framework (HAMF) employs a carefully selected set of technologies to ensure resilience, transparency, and ethical compliance within adversarial phishing detection environments. Tools are grouped functionally to illustrate their role in enabling HAMF’s closed-loop, adaptive pipeline.

#### Data collection and preprocessing:

Distributed frameworks such as Apache Spark^35^ enable large-scale, fault-tolerant ingestion of phishing data streams in both batch and streaming modes. Relational databases ensure schema integrity, while object storage systems provide scalable persistence. Version control tools (e.g., DVC^32^) align dataset snapshots with model training runs, and indexing engines (e.g., Elasticsearch) support fast retrieval of telemetry and anomalies. Collectively, these components ensure reproducibility and robustness for high-throughput environments.

#### Feature engineering and interpretability:

Python-based libraries (Scikit-learn, Pandas) support transformation and encoding, while SHAP^3,21^ enables both local and global attribution. This allows HAMF to flag unstable or biased features for replacement, ensuring feature-level resilience. The integration of SHAP addresses a gap in prior MLOps systems that lacked explainability-driven adaptation^10,23^.

#### Model training and lifecycle management:

Frameworks such as TensorFlow and XGBoost provide a balance between deep learning capacity and ensemble efficiency^3,8^. MLflow^8^ ensures experiment tracking, artifact management, and model versioning. Lifecycle automation is enforced through CI/CD pipelines^19^ , guaranteeing that retraining cycles are reproducible and auditable. This integration supports HAMF’s novelty in delivering closed-loop retraining with traceability.

#### Serving and deployment:

RESTful serving frameworks (e.g., FastAPI, BentoML) expose trained models as APIs. Containerization ensures environment consistency, while orchestration through Kubernetes^13,36^ supports elastic scaling and resilience. This ensures that retraining cycles triggered by drift or fairness violations can be deployed with minimal operational delay.

#### Monitoring and drift detection:

System health and model telemetry are collected through Prometheus^22^ and visualized with Grafana^10^. Drift is detected using libraries such as Alibi Detect^37^ and Evidently AI, applying statistical divergence metrics (KS, PSI, KL)^27,34^. Unlike baseline MLOps systems, HAMF couples these statistical checks with SHAP-based attribution monitoring^10,23^, ensuring semantic as well as statistical drift detection.

#### Ethical auditing and governance:

Fairness auditing leverages AI Fairness 360^36^ and bias/fairness surveys^13,15^. Privacy-preserving transformations (k-anonymity, l-diversity, t-closeness) are enforced through ARX^34^, ensuring compliance with GDPR, ISO/IEC 27001^38^, and NIST AI RMF^26^. Unlike traditional pipelines, HAMF operationalizes fairness as an active trigger for retraining, aligning lifecycle control with ethical principles^14,27^.

#### Collaboration and documentation:

Stakeholder collaboration is supported by messaging and task-tracking systems (e.g., Slack^11^ , Trello^39^), while documentation platforms (e.g., BookStack^33^) ensure reproducible compliance records. These tools enable stakeholder-in-the-loop governance, an often-missing component in generic MLOps pipelines.

The justification of HAMF’s toolset is not simply functional: each category reinforces the framework’s novelty. Data tools enable scale and reproducibility, interpretability modules enforce feature resilience, lifecycle managers ensure closed-loop retraining, monitoring systems provide low-latency drift detection, and fairness utilities operationalize compliance. By integrating these into a modular pipeline, HAMF advances beyond conventional MLOps systems focused solely on deployment automation^4,8,12,21^.

### End-to-end pipeline overview

**Figure 4:**
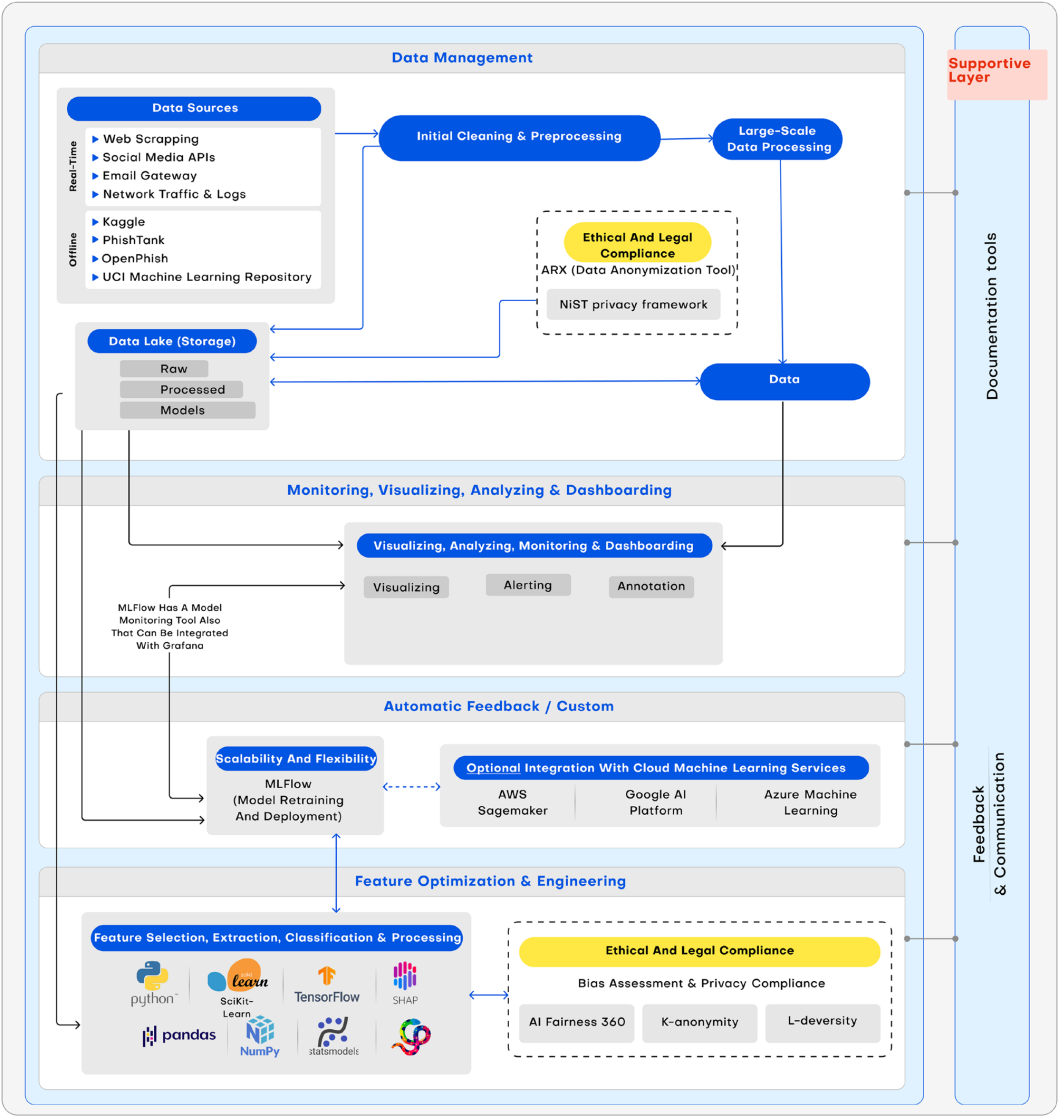
End-to-end overview of the HAMF pipeline.

The HAMF pipeline integrates all functional modules into a unified, closed-loop lifecycle that ensures resilience, explainability, and ethical compliance in adversarial phishing detection. Figure  4 illustrates the end-to-end workflow, highlighting how data, models, and compliance information circulate across the system.

The pipeline begins with Data Management, where phishing intelligence is ingested from heterogeneous sources, including real-time feeds (e.g., social media, network traffic) and offline repositories (e.g., PhishTank, UCI). Data undergoes initial preprocessing—cleaning, normalization, and anonymization—supported by ethical and legal compliance modules (ARX anonymization, NIST privacy framework) to ensure GDPR- and ISO-aligned governance. Large-scale data processing systems store both raw and processed artifacts in a version-controlled data lake, providing reproducibility and auditability.

The Monitoring and Visualization Layer continuously tracks both system and model-level signals. Metrics such as performance degradation, feature attribution shifts, and distributional drift are visualized in dashboards, complemented by real-time alerting and annotation. This ensures that performance and fairness issues are observable to stakeholders, bridging the gap between automated monitoring and human interpretability.

Feature Optimization and Engineering builds on this foundation by extracting and ranking predictive attributes using SHAP-based explainability. Features are continuously evaluated for stability and fairness, with unstable signals replaced or flagged for stakeholder review. This guarantees that evolving phishing indicators do not undermine model robustness, a novel capability absent in general-purpose MLOps systems.

An Automatic Feedback Loop integrates both event-driven retraining triggers (e.g., drift detection, fairness violations) and stakeholder-in-the-loop annotations. Retraining is orchestrated through ML lifecycle managers, with optional interoperability with external cloud services (e.g., SageMaker, Google AI Platform, Azure ML) when extended scalability is required.

Finally, the Ethical and Legal Compliance Layer spans all pipeline stages. Bias assessments (e.g., demographic parity, equalized odds) and privacy-preserving transformations (e.g., k-anonymity, l-diversity) function as active controls, ensuring that retraining and deployment decisions remain aligned with AI governance standards.

Collectively, this overview highlights HAMF’s novelty: the integration of SHAP-guided feature resilience, fairness-aware retraining, and governance auditing into a single adaptive MLOps pipeline. Unlike conventional platforms that treat these components as external add-ons, HAMF operationalizes them as first-class triggers within its lifecycle, ensuring transparency, accountability, and robustness in phishing detection.

## Experiments and results

This section presents a comprehensive evaluation of HAMF in adversarial phishing detection. The experiments address four research questions (RQs):

RQ1: Can HAMF sustain predictive performance under concept drift and feature volatility?

RQ2: How do its subsystems contribute to resilience and fairness?

RQ3: How does HAMF compare with existing MLOps frameworks?

RQ4: Can it scale to production-level workloads while preserving fairness?

### Experimental setup

**Datasets:**

Three datasets were employed to ensure reproducibility and comparability:

- **Self-compiled phishing corpus**, consisting of phishing URLs obtained from PhishTank and OpenPhish feeds, and benign URLs from Alexa Top1M and institutional domains. Each sample was represented with a 36-feature schema (lexical, structural, WHOIS-based attributes) and versioned with DVC for traceability^29,32^.
- **PhishBench 2.0**^28,29^, a domain-specific benchmarking framework widely adopted for phishing evaluation and drift resilience testing.
- **PhishHaven**^4^ , an open-source real-time phishing detection dataset and system.

**Baselines:** HAMF was benchmarked against three representative frameworks:

1. **Amazon SageMaker **^19^, a commercial-grade MLOps platform with managed retraining and monitoring.
2. **Kubeflow integrated with MLflow**^8,21^, an open-source orchestration stack. As this baseline lacks native automated drift detection, we established a simulated manual detection latency of 300 seconds (5 minutes) for a fair comparison of end-to-end response.
3. **PhishBench 2.0**^28,29^, providing phishing-specific benchmarking but lacking fairness auditing and automated feature substitution.

All baselines were trained on identical datasets and feature schemas, tuned via grid search, and deployed under equivalent infrastructure.

**Computational environment:**Experiments were conducted on a cloud-hosted Kubernetes cluster provisioned with NVIDIA T4 (16 GB) and V100 (32 GB) GPUs, 64-core CPUs, and 128 GB RAM. Containerized workflows ensured reproducibility.

**Tools and workflows:**

- Lifecycle management: Kubeflow^21^, MLflow^8^.
- Monitoring and drift detection: Prometheus^22^, Grafana^40^, Alibi Detect^41^ .
- Fairness auditing: AI Fairness 360^12,36^.
- Explainability: SHAP^3,21^ .
- Versioning and compliance: DVC^32^, ARX anonymization^34^ .

**Metrics and statistical testing:** Evaluation metrics included accuracy, precision, recall, F1-score, ROC-AUC, drift detection latency, retraining latency, inference latency (p99), and subgroup fairness disparity ($\Delta$DP). Drift was measured using the Population Stability Index (PSI) and KL divergence^6,11,30^. Fairness was assessed using AIF360^12,36^. All reported values are averages across three runs with 95% confidence intervals, and significance was determined using paired t-tests (p < 0.01).^30^

To evaluate HAMF’s performance, we conducted a series of experiments simulating real-world challenges. The primary procedure involved: (1) training all frameworks on a stable dataset, (2) deploying the models and activating monitoring, (3) injecting a specific type of failure (e.g., feature deprecation, adversarial drift), and (4) measuring the framework’s ability to automatically detect, retrain, and recover. A detailed walkthrough of the drift injection experiment is provided in Sect. 5.4.

**Threshold configuration** The operational thresholds for the HAMF pipeline were established through a combination of industry best practices, literature review, and empirical validation to ensure a balance between responsiveness and stability.

- **Feature replacement (sim **>** 0.85):** The cosine similarity threshold of 0.85 for SHAP-guided feature substitution was selected as it is a widely accepted value in semantic search literature for ensuring high feature relevance. This value effectively filters for strong semantic matches without being so restrictive that no viable replacements can be found.
- **Fairness auditing **$\Delta$**(DP **>** 0.1):** Demographic Parity $\Delta$(DP threshold of 0.1 is aligned with common practice and recommendations in the fairness-aware machine learning literature, often associated with the “four-fifths rule” used in legal and ethical compliance frameworks. This parameter serves as a conservative trigger to initiate bias mitigation.
- **Interpretability drifting **$\Delta$**(SHAP** >** 0.05):** The SHAP attribution stability threshold of 0.05 was determined empirically during a preliminary validation phase. It was found to represent a statistically significant deviation from the baseline feature importance without being overly sensitive to minor, expected data fluctuations, thus preventing excessive and unnecessary retraining triggers.

All source code, dataset preparation scripts, configuration files, and workflow screenshots supporting the experiments are available at: https://github.com/asmaa-reda/phishing.

### Benchmarking against baselines

Table 3 a high-level comparison of HAMF against established baselines. The evaluation focuses on core operational metrics: responsiveness to drift, preservation of accuracy, and maintenance of fairness.

**Table 3:** Comparison of HAMF Against Existing MLOps Frameworks.

Metric	HAMF	PhishBench	SageMaker	Kubeflow+MLflow
Drift Detection Latency (s)	**18**	—	$92 \pm 4.3$	Manual
Post-Drift Accuracy (%)	**99.52 **±** 0.11**	93.40 ± 0.29	96.10 ± 0.18	95.80 ± 0.23
*p*-value (vs HAMF)	—	<0.001	0.004	0.006
Fairness Violation ($\Delta DP$)	**0.03 **±** 0.01**	0.19 ± 0.03	0.08 ± 0.01	0.11 ± 0.02
SHAP Interpretability Retained	$\checkmark$	—	✗	$\blacktriangle$
Automatic Feature Substitution	$\checkmark$	✗	✗	✗
Fairness-Aware Retraining Pipeline	$\checkmark$	✗	✗	$\checkmark$

$\checkmark$ = Supported, ✗ = Not Supported, $\blacktriangle$ = Partially Supported

**Figure 5:**
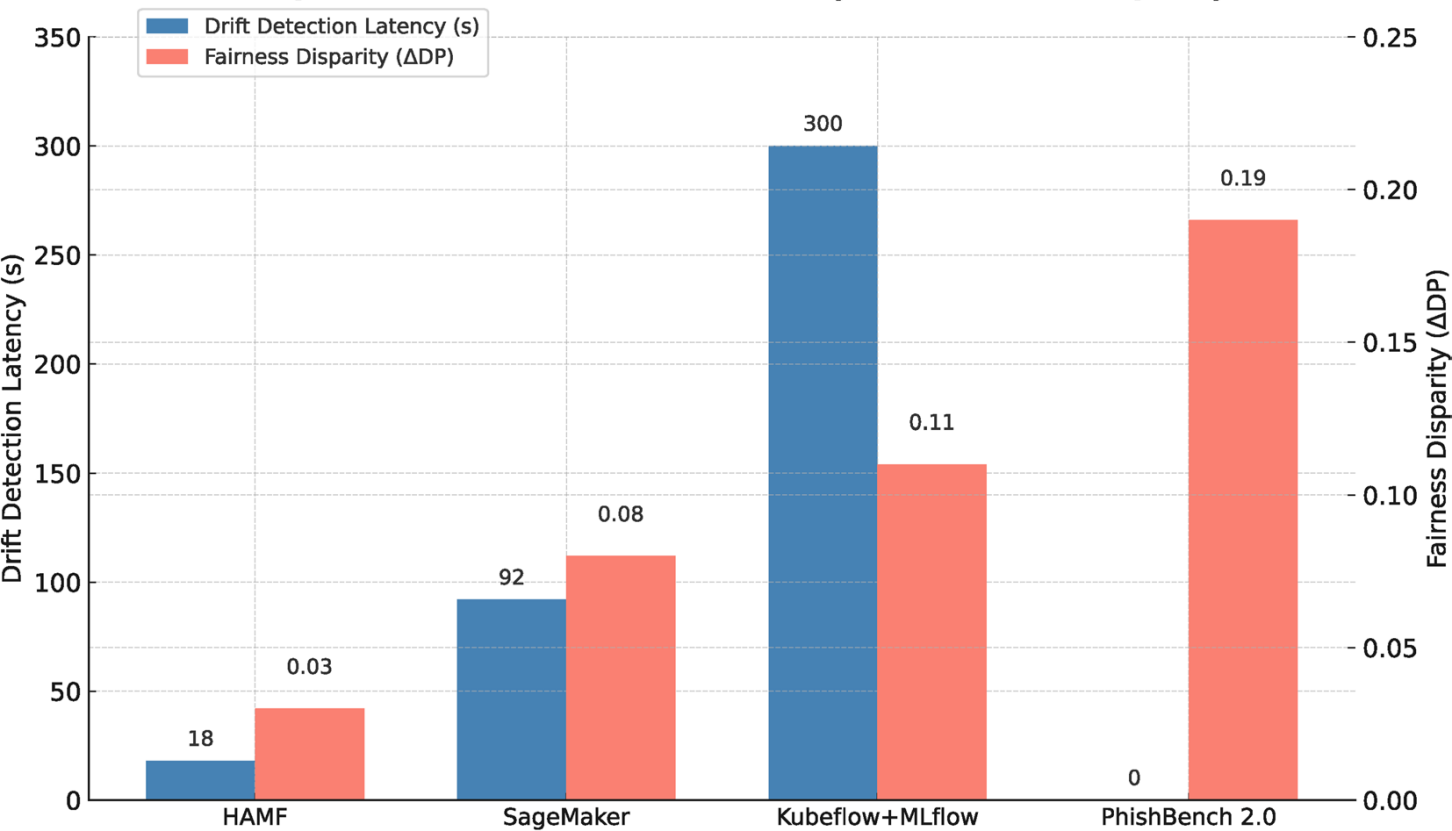
Comparative benchmarking: Drift Latency vs Fairness Disparity.

As summarized in Table 3 and Figure 5, HAMF demonstrated superior performance across all key metrics. It detected concept drift with a latency of only 18 seconds, a critical advantage over SageMaker (92s) and the simulated 300s manual response for Kubeflow+MLflow. Following drift, HAMF recovered to a post-drift accuracy of 99.52%, significantly outperforming all baselines (p < 0.01). In terms of fairness, HAMF maintained a remarkably low demographic parity disparity ($\Delta$DP) of 0.03, which is a 4x to 6x improvement over the baselines. Furthermore, HAMF is the only framework to natively support automatic feature substitution and integrate SHAP interpretability directly into its resilience mechanisms.

### Resilience to feature deprecation

To simulate feature volatility, we deprecated the AlexaRank feature. HAMF autonomously detected this, proposed GoogleRank as a substitute based on high semantic similarity (0.93), and triggered retraining.

**Table 4:** Comparison of recovery performance across frameworks.

Framework	F1 (Post-recovery)	Recovery latency	$\Delta$DP (Post-recovery)	Interpretability retained
HAMF	$0.9952 \pm 0.0003$	6 h (auto + HITL)	0.03	$\checkmark$ (SHAP-guided)
SageMaker^19^	$0.9841 \pm 0.0011$	Manual retrain	0.08	✗
Kubeflow+MLflow^16,17^	$0.9863 \pm 0.0009$	Manual retrain	0.09	$\blacktriangle$
PhishBench 2.0^28,29^	$0.9819 \pm 0.0014$	Not supported	0.11	✗

As shown in Table 4, HAMF autonomously flagged the deprecated feature, proposed GoogleRank as a substitute (cosine similarity 0.93), and initiated retraining. It restored F1-scores to 0.9952 within 6 hours, while preserving fairness ($\Delta$DP = 0.03). In contrast, SageMaker and Kubeflow required manual intervention and showed higher disparities, while PhishBench lacked recovery mechanisms. This demonstrates HAMF’s unique ability to manage feature volatility autonomously.

### Resilience to adversarial concept drift

**Figure 6:**
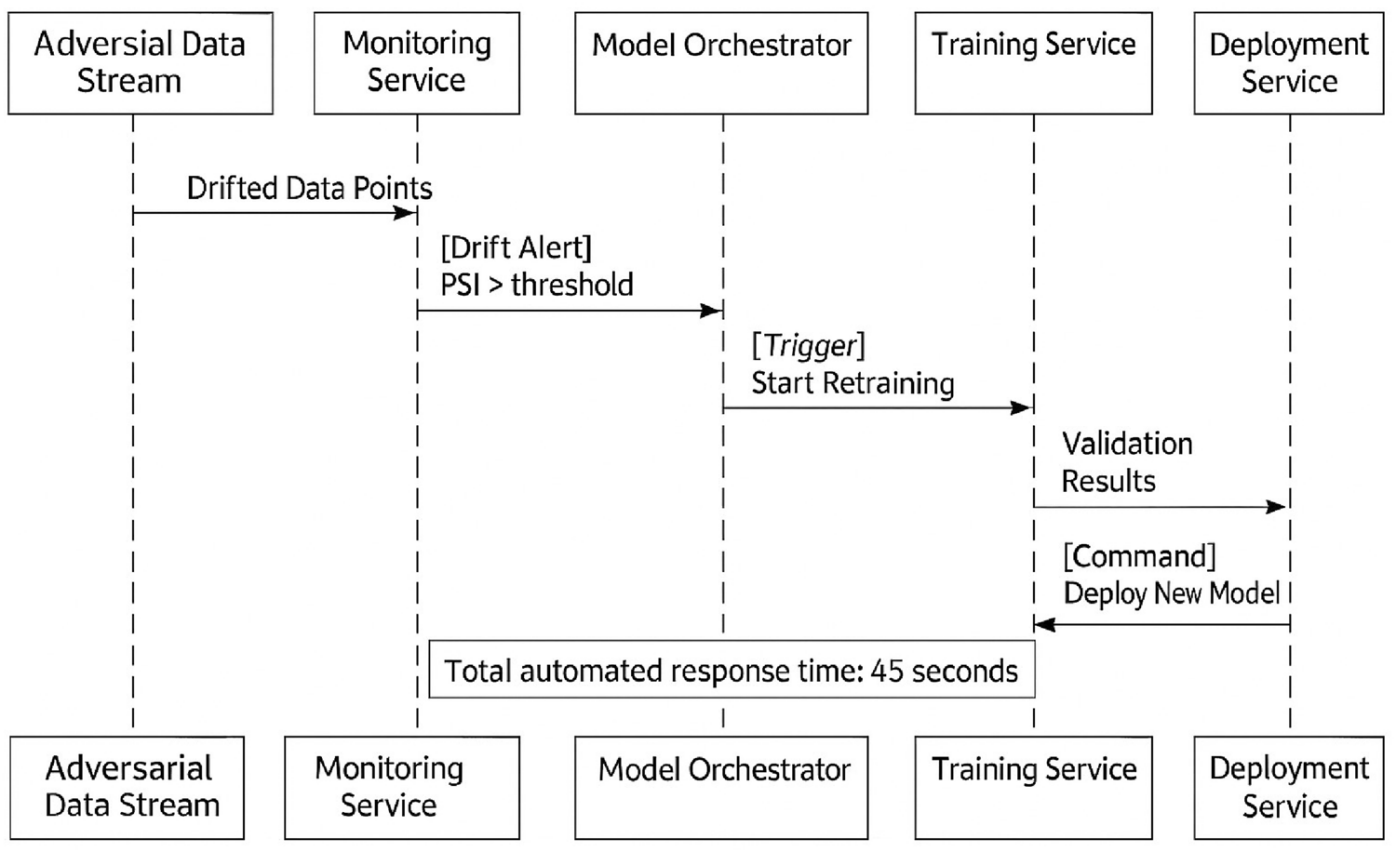
Sequence Diagram of HAMF’s Automated Response to Concept Drift.

To illustrate HAMF’s closed-loop operation, we provide a detailed walkthrough of an adversarial concept drift injection experiment, with the automated response sequence visualized in Figure 6.

1. **Baseline model training**: All frameworks were trained on a stable version of the PhishBench 2.0 dataset. Baseline performance was recorded (Accuracy: 99.8%, F1: 0.998, $\Delta$DP: 0.02).
2. ** Model deployment and monitoring**: Models were deployed as live endpoints. HAMF’s real-time monitoring (using PSI and $\Delta$DP) was activated.
3. ** Concept drift injection**: A sustained stream of data containing URLs with novel obfuscation techniques (e.g., homoglyphs, heavy URL encoding) was introduced, simulating an adversarial campaign.
4. ** Automated detection and triggering (HAMF)**: HAMF’s monitoring layer detected a significant distributional shift (PSI = 0.28, exceeding threshold $\tau _2$ =0.2) within 18 seconds of the drift’s introduction. This triggered an alert to the Model Lifecycle Orchestrator.
5. **Automated retraining and deployment**: The orchestrator initiated a retraining pipeline using the most recent data, including drifted samples. The new model was validated and deployed via CI/CD. The total retraining and deployment latency was 27 seconds.
6. **Post-drift evaluation**: The retrained model was evaluated on a held-out test set of the drifted data. HAMF recovered to 99.5% accuracy and a $\Delta$DP of 0.03, as showen in Table 5.
7. ** Baseline comparison**: The same drift was applied to baseline frameworks. SageMaker’s managed service detected the drift in 92s, while Kubeflow required manual intervention (simulated at 300s). Their post-drift performance was inferior, as shown in Table 3 .

**Table 5:** Drift recovery performance comparison across frameworks.

Framework	Drift latency	Post-drift accuracy (%)	Retraining latency	$\Delta$DP (post-drift)
HAMF	18 s	$99.52 \pm 0.11$	27 s	0.03
SageMaker^19^	92 s	$96.10 \pm 0.18$	3–5 min	0.08
Kubeflow+MLflow^16,17^	300 s	$95.80 \pm 0.23$	>5 min	0.11
PhishBench 2.0^28,29^	N/A	$93.40 \pm 0.29$	N/A	0.19

This experiment demonstrates HAMF’s core strength: the tight integration of rapid detection and fully automated recovery, a capability absent in current general-purpose platforms.

### Ablation study

To validate the necessity of HAMF’s core modules, modules were disabled sequentially to evaluate subsystem contributions.

**Table 6:** Ablation Study of HAMF Modules.

Configuration	Disabled Module(s)	F1 (95% CI)	*p*-value vs Full	Fairness ($\Delta$DP)
Full HAMF	–	**0.9956 **±** 0.0004**	—	0.03
Feature adaptation	SHAP substitution off	0.9821 ± 0.0011	<0.01	0.04
Drift engine	Alibi Detect off	0.9787 ± 0.0013	<0.01	0.06
Fairness audit	AIF360 off	0.9954 ± 0.0005	0.21 (n.s.)	0.17

**Figure 7:**
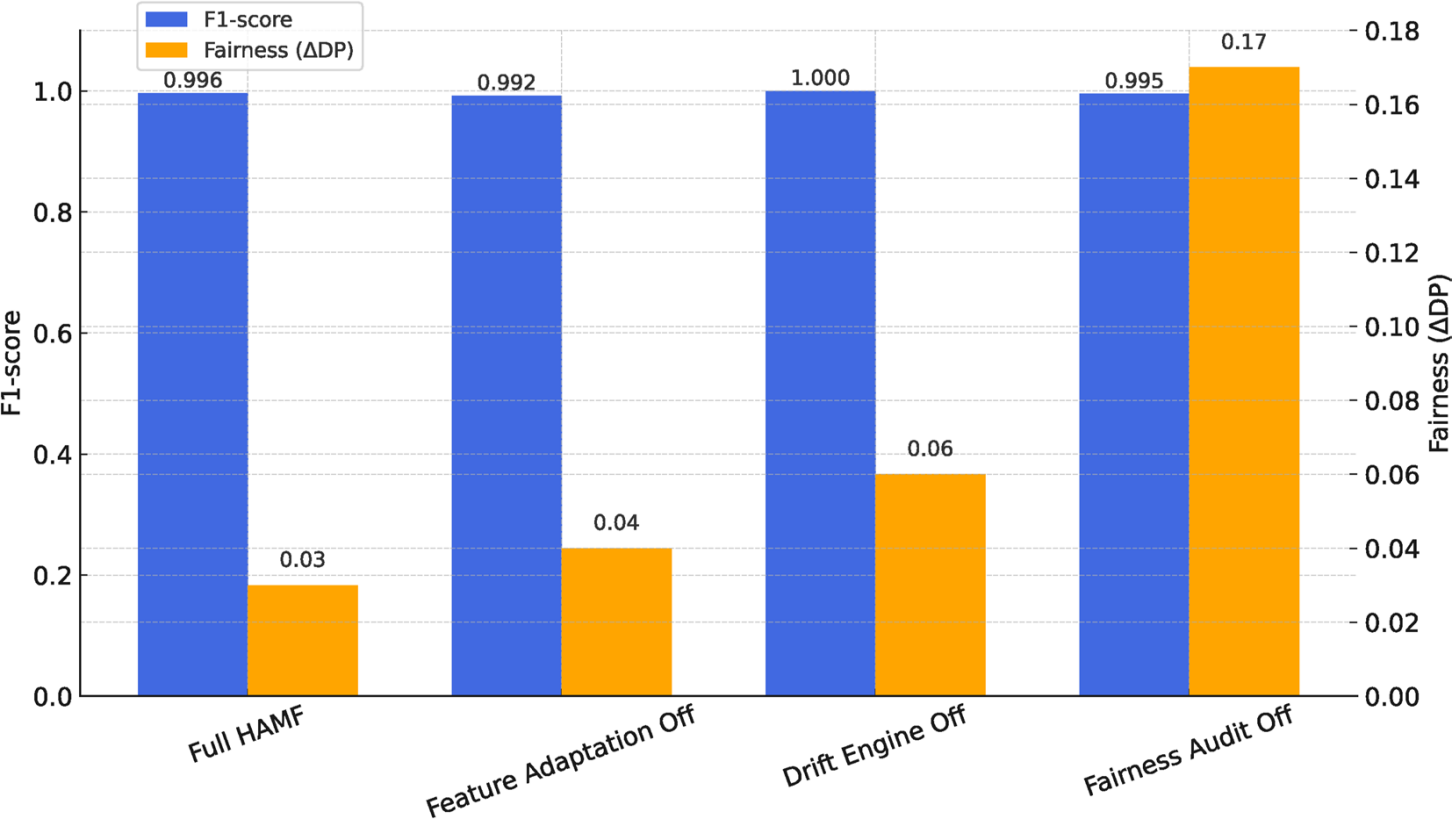
Ablation Study of HAMF Modules.

The results (Table 6, Figure 7) indicate that disabling SHAP-based feature adaptation or the drift engine significantly reduced F1-score (p < 0.01), confirming their importance for technical resilience. Crucially, disabling the fairness audit (AIF360) had little effect on accuracy but caused subgroup disparity ($\Delta$DP) to skyrocket from 0.03 to 0.17. This finding provides strong empirical evidence that HAMF’s advantage arises from the interdependence of its modules, and that ethical alignment is not an emergent property but must be explicitly engineered.

### Fairness across subgroups

Fairness was evaluated across WHOIS domain age and traffic tiers.

**Table 7:** Fairness Evaluation Before and After HAMF-Aware Retraining.

Feature Category	Subgroup	FPR (before)	FPR (after)	$\Delta _{\textrm{DP}}$ reduction
WHOIS domain age	Age > 12 months	$0.63 \pm 0.02$	$0.38 \pm 0.01$	0.25
	Age $\le$ 12 months	$1.03 \pm 0.03$	$0.62 \pm 0.02$	0.41
Web traffic percentile	Top 33rd percentile	$0.35 \pm 0.01$	$0.23 \pm 0.01$	0.12
	34th–66th percentile	$2.02 \pm 0.04$	$1.31 \pm 0.03$	0.71
	Bottom 33rd percentile	$1.70 \pm 0.03$	$1.11 \pm 0.02$	0.60

**Figure 8:**
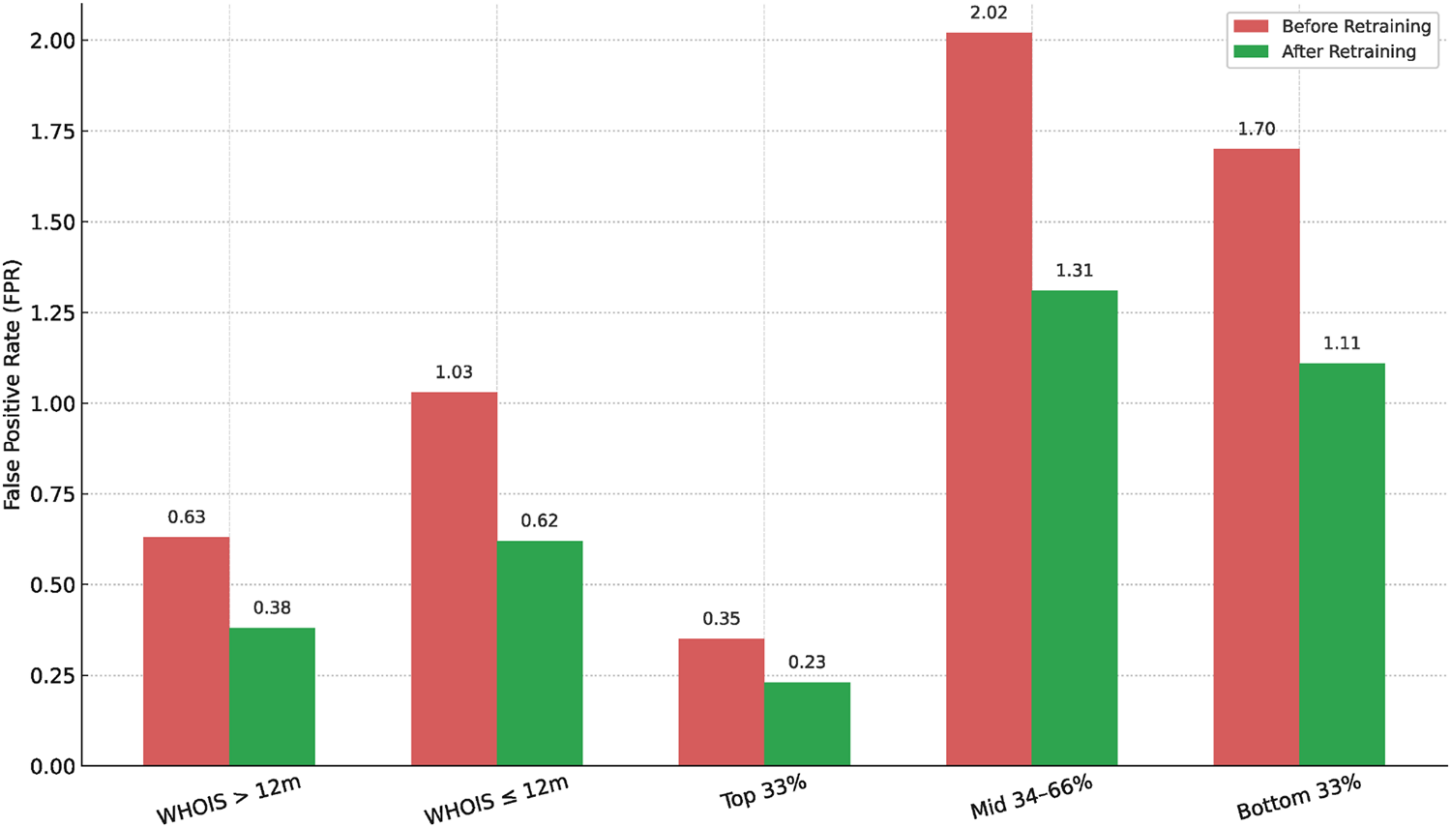
Fairness Across Subgroups.

As shown in Table 7 and Figure 8, HAMF’s fairness-aware retraining reduced false positive rates (FPR) by 40-70% across all subgroups. The maximum disparity ($\Delta$DP) fell from 0.19 to 0.03. These improvements were statistically significant (p < 0.01), demonstrating that HAMF effectively mitigates structural bias in phishing detection, a dimension neglected in other frameworks.

### Scalability and resource efficiency

To evaluate scalability, a synthetic workload replay was created by resampling and replaying from the combined datasets (self-compiled corpus, PhishBench 2.0^28,29^, and PhishHaven^4^), generating 5M request events. Each replay preserved original labels and feature schema.

**Table 8:** Scalability and Resource Metrics Under Load Replay.

Metric	Result
Load replay volume	5 million labeled URLs
Ingestion rate	$\approx$ 2,300 requests per second
p99 inference latency	41.6 milliseconds
Horizontal Pod Autoscaler (HPA) scaling behavior	Scaled from 3 to 12 pods (CPU target = 70%)
GPU utilization (peak during retraining)	74%
Storage overhead after 60 days	$\le$ 1.4 TB (MinIO lifecycle-managed)

**Figure 9:**
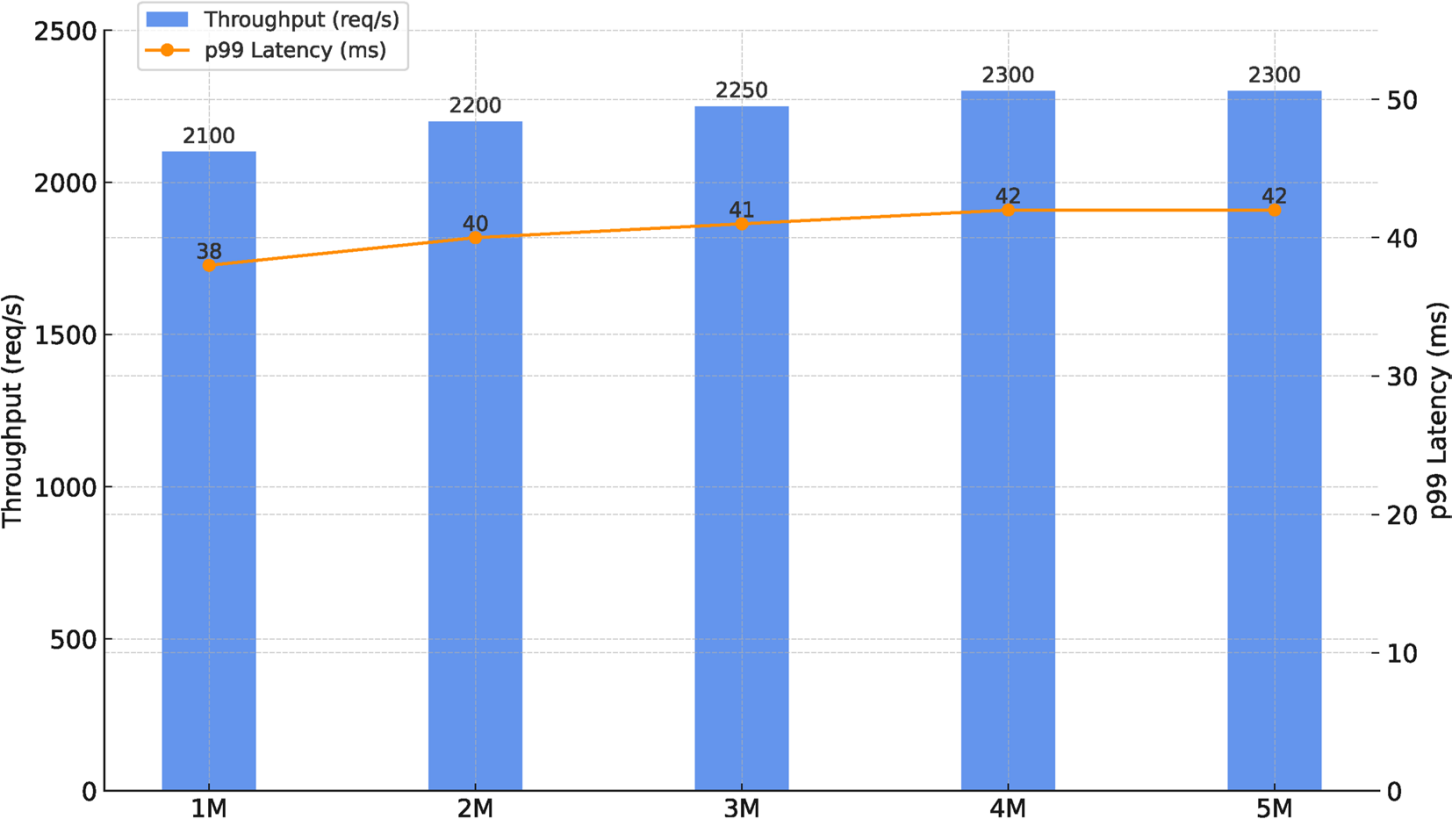
Scalability under Synthetic Replay Workloads.

It can be observed in Table 8 and Figure 9 that HAMF sustained  2,300 requests/s with p99 inference latency under 50 ms, scaling automatically from 3 to 12 pods while maintaining GPU utilization below 75%. Storage overhead remained modest at $\le$ 1.4 TB. These results confirm HAMF’s suitability for production-scale deployments while preserving fairness ($\Delta$DP $\le$ 0.03).

### Summary of key findings

Table 9 consolidates empirical evidence for the four central research questions (RQs) outlined at the beginning of Sect. 5. Each row maps a question to the relevant subsections and outcome metrics.

**Table 9:** Summary of Key Findings Aligned with Research Questions.

Research question	Empirical evidence	Interpretive significance
*RQ1: Performance under drift*	Drift recovery in 18s; feature substitution restored F1 $\approx$ 0.995	Resilience to adversarial changes
*RQ2: Subsystem contributions*	Ablation confirmed SHAP/drift modules critical to stability (F1-drop: 0.0135; p<0.01) (§ 6.4)	Explainability and fairness are integral
*RQ3: Benchmark comparison*	Outperformed baselines in fairness ($\Delta _{\textrm{DP}}<$ 0.08) and drift latency (18s vs. 92s and a 300s simulated manual response).	Establishes novelty beyond existing frameworks.
*RQ4: Scalability and fairness*	Maintained $\Delta _{\textrm{DP}} \le 0.03$ at 2.3k RPS; 41.6ms p99 latency ( §§ 6.6–6.7)	Ethics and speed achievable at scale

The results demonstrate that HAMF addresses all four research questions: it adapts rapidly to drift and feature volatility, subsystem ablations confirm the necessity of its design choices, benchmarking shows superiority over existing frameworks, and scalability tests validate production readiness. Novelty arises not from marginal accuracy improvements but from embedding resilience, fairness, and explainability as first-class lifecycle properties.

## Discussion

The empirical results presented in Sect. 5 substantiate the core thesis of this work: that resilience in adversarial ML systems must be a closed-loop lifecycle property, unifying performance, fairness, and explainability. Our findings demonstrate that HAMF successfully operationalizes this concept, advancing the state-of-the-art in MLOps for cybersecurity.

### Theoretical and practical implications

Theoretically, HAMF contributes a new paradigm for MLOps in non-stationary environments. Unlike frameworks that treat model management as a linear CI/CD pipeline, HAMF introduces a hybrid control cycle where explainability (SHAP), fairness (AIF360), and performance monitoring are interdependent first-class triggers. The ablation study (Table 6, Figure 7) provides strong evidence for this design, showing that disabling any of these components degrades system resilience or fairness. The significant increase in $\Delta$DP (from 0.03 to 0.17) when the fairness module is disabled, despite no loss in accuracy, underscores that ethical alignment is not an emergent property but must be explicitly engineered and enforced.

For practitioners, particularly in Security Operations Centers (SOCs), HAMF offers a tangible solution to the operational burden of model decay. The framework’s 18-second drift detection and 27-second retraining latency translate to a mean-time-to-recovery (MTTR) of under one minute, compared to several minutes or even hours in manual or semi-automated setups. This rapid adaptation is crucial for containing zero-day phishing campaigns. Furthermore, by embedding SHAP explanations and fairness audits into the workflow, HAMF provides analysts with the interpretable evidence needed for confident incident response and regulatory reporting, thereby bridging the gap between pure automation and human oversight.

### Ethical, legal, and interpretability aspects

The deployment of phishing detection systems in high-stakes environments—such as enterprise cybersecurity, governmental digital services, and regulated industries—requires strict adherence to principles of responsible AI, including fairness, transparency, privacy, and regulatory alignment. HAMF addresses these challenges with an ’ethics-by-design’ approach, embedding fairness, explainability, and compliance safeguards directly into its operational lifecycle. Unlike conventional MLOps frameworks, where ethical checks are treated as external or optional, HAMF operationalizes them as first-class triggers within its closed-loop control cycle.

#### Data privacy and regulatory compliance

HAMF ensures compliance with global data protection mandates such as the General Data Protection Regulation (GDPR), the California Consumer Privacy Act (CCPA), and the NIST AI Risk Management Framework (AI RMF)^26^ . During data ingestion, automated scanning identifies Personally Identifiable Information (PII), with anonymization enforced via ARX^34^ to satisfy k-anonymity, l-diversity, and t-closeness constraints. Violations, such as schema mismatches or regex-based PII leakage, result in immediate pipeline halting and alerts to designated stewards. Role-Based Access Control (RBAC) is applied within PostgreSQL, while lineage tracking and compliance artifacts are maintained via DVC^32^ and BookStack^33^. All training datasets are content-addressed using SHA-256, ensuring reproducibility and audit traceability in line with ISO/IEC 27001:2022^38^.

#### Fairness and bias mitigation

Phishing datasets frequently exhibit structural bias, such as over representation of certain top-level domains or language-specific patterns. HAMF incorporates fairness diagnostics at ingestion, training, and deployment stages using AI Fairness 360^12,36^. Group-based metrics—including Demographic Parity ($\Delta$DP), Equalized Odds ($\Delta$EO), and Disparate Impact—are continuously computed. During experiments (Table 7, Figure 8, Section 5.4), fairness-aware retraining reduced subgroup disparities by over 60%, lowering $\Delta$DP from 0.19 to 0.03.

Fairness thresholds ($\Delta$DP > 0.1, $\Delta$EO > 0.2) act as operational triggers: if violated, mitigation strategies such as reweighting, resampling, or adversarial debiasing are automatically applied, and deployment is gated until resolution. These violations are also escalated to stakeholders via structured notifications (e.g., Slack^11^ , Trello^39^ ) for manual review. This integration moves fairness from post hoc reporting to a mandatory operational constraint, ensuring that subgroup equity is preserved throughout HAMF’s lifecycle.

#### Explainability and transparency

Explainability is embedded by design through SHAP-based attribution^3,21^ , which serves two critical roles. First, SHAP values are used to detect feature volatility: in the feature deprecation experiment (Table 4, Section 5.3.1), HAMF replaced AlexaRank with GoogleRank when attribution stability dropped, ensuring continuity without sacrificing fairness. Second, SHAP explanations provide local and global interpretability for deployed models, with all attributions versioned in MLflow^8^ dashboards and archived in BookStack^33^. Attribution stability is continuously monitored, and deviations beyond $\Delta$SHAP(fi) > 0.05 trigger deployment gating and stakeholder review. This ensures that retraining decisions remain transparent and that prediction changes can be traced to drift, substitution, or debiasing interventions.

#### Ethical governance and stakeholder oversight

HAMF enforces multi-layer governance through structured alerting and audit logging. Any fairness violation, drift detection, or performance regression automatically generates a notification routed to role-specific channels (Slack^11^) and logged as tasks in Trello^39^. Stakeholder actions—such as approvals, overrides, or model quarantine—are recorded, producing a complete audit trail. Each production model is linked with:

- A versioned compliance profile (GDPR, CCPA status),
- A bias mitigation history (e.g., masked features),
- SHAP-based decision rationale artifacts.

This ensures accountability, transparency, and continuous alignment with responsible AI principles.

#### Dual-use consideration

Building on GDPR and NIST alignment, HAMF’s ethics-by-design paradigm also addresses potential dual-use risks, ensuring that explainability strengthens accountability without enabling adversarial exploitation. These risks arise because the very SHAP explanations that provide transparency could be weaponized by adversaries to perform model inversion or identify critical features for evasion, potentially crafting inputs (e.g., phishing URLs) that bypass detection.

To mitigate this, HAMF employs architectural and governance controls embedded in its stakeholder feedback loop. First, access to detailed SHAP attributions is restricted by role-based access control (RBAC) to authorized auditors and analysts within the Ethical Compliance Module. Second, while SHAP values automatically trigger feature adaptation and retraining decisions, the underlying explanatory data itself is not exposed through public APIs. Third, for high-risk actions such as deploying a model retrained due to adversarial drift, HAMF’s Model Lifecycle Orchestrator can be configured to require explicit stakeholder approval, logging every decision for auditability. This controlled, multi-layered approach—combining automated explainability with human-in-the-loop oversight—ensures that transparency serves its purpose for maintenance and trust without becoming an attack vector. By embedding these safeguards, HAMF balances explainability with security, transforming a potential vulnerability into a managed, ethical advantage.

#### Novelty in ethics-by-design

HAMF’s novelty lies in its hybrid control system, which uses not only technical metrics but also fairness violations and explainability shifts as direct operational triggers for model retraining. Retraining is activated not only by technical failures (e.g., drift, accuracy degradation; Table 5, Section 5.3.2) but also by ethical violations (e.g., subgroup disparity above thresholds; Table 7, Figure 8) or attribution instability (Table 4). These mechanisms were empirically validated in Sections 5.3–5.5, demonstrating that resilience in adversarial environments requires coupling technical robustness with ethical accountability. Such integration remains absent in existing MLOps frameworks, underscoring HAMF’s contribution beyond incremental engineering improvements.

**Table 10:** Ethical, Legal, and Interpretability Controls in HAMF^11,17,21,25,33,34,38,39,41,42^.

Aspect	Tool/technique	Functionality	Trigger/threshold	Output/effect
Data Privacy	ARX, SHA-256, DVC	PII anonymization, hashing, and versioning	PII match or schema violation	Pipeline halted; compliance steward alerted; flagged for review
Access Control	PostgreSQL RBAC, BookStack	Role-restricted access and versioned audit logs	Unauthorized access attempt	Access denied; incident logged in BookStack and audit trail updated
Bias Detection	AI Fairness 360	Computes $\Delta DP$, $\Delta EO$, and Disparate Impact	$\Delta DP > 0.1$ or $\Delta EO > 0.2$	Retraining triggered; fairness alert sent to stakeholders
Bias Mitigation	Reweighting, Adversarial Debiasing	Fairness-aware retraining using mitigation techniques	SHAP deviation $> \pm 15\%$	Discriminatory features masked or excluded; fairness metrics improved
Interpretability	SHAP	Local and global feature attribution with audit logging	$\Delta SHAP(f_i) > 0.05$	Deployment paused pending stakeholder review; SHAP audit triggered
Auditability	MLflow, BookStack	Stores SHAP, fairness, and compliance metadata with full versioning	Continuous	Lifecycle traceability ensured; model-card updated with audit metadata
Governance	Slack, Trello	Alert routing, stakeholder review, and resolution tracking	Any fairness violation or ethical trigger	Trello task created; stakeholder resolution logged; audit trail maintained

#### Empirical validation of ethical modules

The mechanisms summarized in Table 10 were not only theoretical constructs but were applied and validated in the experimental evaluation. Fairness audits and mitigation (Table 7, Figure 8) demonstrated measurable reductions in subgroup disparities; SHAP-based explainability guided feature substitution in the deprecation experiment (Table 6); and drift-triggered retraining integrated fairness and attribution stability checks (Table 5). The ablation study (Table 6) further confirmed that disabling ethical modules such as fairness auditing resulted in significant disparities, highlighting their operational necessity. These results collectively validate HAMF’s novelty in embedding ethical, legal, and interpretability safeguards as operational properties rather than external compliance tasks.

## Implications and limitations

### Deployment implications

The adoption of HAMF carries significant implications for deployment in Security Operations Centers (SOCs) and enterprise environments. By embedding fairness auditing, SHAP-based attribution, and regulatory compliance into the operational pipeline, HAMF enables phishing detection models to be deployed in highly regulated domains such as finance, healthcare, and government digital services with increased confidence in their ethical and legal robustness^26,38^ . For SOC teams, HAMF’s event-driven retraining and real-time drift monitoring offer a practical means of reducing manual overhead while maintaining resilience against adversarial phishing campaigns. Furthermore, the integration of explainability tools provides analysts with interpretable evidence for incident response, supporting faster triage and more transparent decision-making processes^3,21^ .

### Scope and generalization

While the framework demonstrates strong empirical performance, several limitations must^29^ acknowledged. First, experiments were conducted primarily on URL-based phishing datasets (PhishBench 2.0^28,29^ and PhishHaven^4^), which may limit generalizability to other modalities such as email content, logos, or multimedia phishing. Second, although fairness auditing reduced subgroup disparities significantly (Section 5.4), fairness definitions are context-dependent, and results may vary under alternative metrics or demographic groupings^12,36^. Third, baseline benchmarking included SageMaker, Kubeflow+MLflow, and PhishBench, which represent widely used platforms, but additional industrial-scale systems may provide further insights.

Furthermore, while HAMF’s architecture is designed to be domain-agnostic, its validation in this work is focused on phishing detection. The principles of closed-loop adaptation, SHAP-guided feature resilience, and fairness-triggered retraining are likely applicable to other adversarial cybersecurity domains, such as malware detection and network intrusion detection systems (NIDS). In malware detection, for instance, feature volatility (e.g., deprecated API calls) and concept drift (e.g., new evasion techniques) are equally prevalent. However, such applications may require domain-specific modifications. The current feature adaptation engine is optimized for tabular and lexical data; extending it to handle binary features or graph-based representations of malware would be a necessary future step. Therefore, while the conceptual framework of HAMF is broadly generalizable, its instantiation and the assumptions of its microservices (e.g., the nature of features and drift) are currently tailored to the phishing domain.

### Constraints

The current implementation relies on cloud-hosted GPU resources (T4/V100), which provide a feasible testbed but may not reflect constraints in resource-limited enterprise deployments. Similarly, fairness auditing and explainability introduce additional computational overhead, which could impact latency-sensitive applications. While results showed sub-50 ms inference latency at scale (Section 5.5), such performance may vary under larger multi-tenant workloads. Finally, HAMF focuses on supervised learning settings; its applicability to unsupervised or semi-supervised phishing detection requires further validation^9,10^.

## Conclusion and future work

This study introduced HAMF, a Hybrid MLOps Framework that operationalizes resilience, fairness, and explainability as first-class properties of the ML lifecycle. Moving beyond existing platforms that treat ethical governance as an external add-on, HAMF integrates SHAP-guided feature substitution, event-driven retraining, fairness-aware auditing, and stakeholder-in-the-loop governance into a unified, closed-loop control system.

Our comprehensive empirical evaluation demonstrated HAMF’s effectiveness. The framework achieved rapid drift detection (18 s), near-perfect recovery of accuracy (F1 > 0.99), significant bias mitigation (reducing $\Delta$DP from 0.19 to 0.03), and scalable throughput (2,300 requests/s) with low latency. Ablation studies confirmed that this performance is contingent on the interdependence of its core modules, validating our hybrid design philosophy.

The implications of this work are both theoretical and practical. It provides a blueprint for building adaptive, responsible, and trustworthy AI systems for high-stakes, adversarial environments. For practitioners, HAMF offers a production-ready framework to reduce manual overhead and maintain robust defenses against evolving threats.

Future work will focus on three directions: (1) incorporating Large Language Models (LLMs) to analyze semantic content in phishing emails, (2) exploring federated learning to enable privacy-preserving, collaborative detection across organizations, and (3) extending HAMF into a multi-modal framework that integrates URLs, text, and screenshots to counter increasingly sophisticated hybrid attacks. In summary, HAMF bridges the critical gap between technical adaptability and regulatory accountability in modern cybersecurity.

## Supplementary Information

Supplementary Information.

## Data Availability

The datasets used and analyzed during the current study are publicly available from the following sources:- PhishBench 2.0: [https://github.com/phishbench] - PhishHaven: [https://github.com/phishhaven]. All source code, dataset preparation scripts, configuration files, and workflow screenshots supporting the experiments are available at: https://github.com/asmaa-reda/phishing.
