# Supplementary material for: Hybrid MLOps framework for automated lifecycle management of adaptive phishing detection models
Source: Sci Rep. 2025 Nov 4;15:38478. doi: 10.1038/s41598-025-23600-z (PMC12586440; doi:10.1038/s41598-025-23600-z)
Supplement: Supplementary file 1 — Supplementary Information. [file 41598_2025_23600_MOESM1_ESM.pdf]

Supplementary Information for:

Hybrid MLOps Framework for Automated Lifecycle Management of Adaptive Phishing Detection Models

Asmaa Reda, Shereen Taie, Masoud E. Shaheen

Supplementary Note 1: Computational Complexity Analysis

Time Complexity

Let:

- n = number of data records per batch
- f = number of active features
- k = number of preprocessing transformations
- m = number of monitored metrics or SHAP checks
- e = number of training epochs or estimator iterations
- s = number of microservices in deployment
- v = number of tracked model or dataset versions

The total time complexity is:

$$\begin{aligned} T_{\text{HAMF}} &= O(n \cdot k + f \cdot n + f \cdot \log f + m \cdot n + n \cdot f \cdot e + s \cdot \log v) \\ &= O\big(n \cdot (k + f + m + f \cdot e) + f \cdot \log f + s \cdot \log v\big) \end{aligned} \tag{1}$$

Note: The SHAP computation per feature is assumed to be $O(n)$ under tree-based approximation methods...

Space Complexity

Let h denote the historical SHAP tracking window length. Then the space requirements include:

- $O(n)$ for streaming and stored data
- $O(f)$ for active feature metadata
- $O(f \cdot h)$ for SHAP history (e.g., 30-day attribution window)
- $O(s)$ for logs, audit records, and deployment artifacts

The total space complexity is:

$$S_{\text{HAMF}} = O(n + f + s + f \cdot h) = O(n + f \cdot (1 + h) + s) \tag{2}$$

These bounds confirm that HAMF maintains computational tractability...

Supplementary Note 2: Algorithm Pseudocode

Algorithm 1: Algorithm S1: HAMF Core Execution Flow

Input: Incoming data stream S, Initial model M_0, Initial feature set F_0

Output: Deployed models, Monitoring logs, Audit trails

RegisterAssets(M_0, F_0) ; `// Step 1: Model registration`
$D_{ref} \leftarrow$ LoadReferenceData() ; `// GDPR-compliant reference baseline`
while *system is operational* **do**
  $D_t \leftarrow$ IngestStream(S) ; `// Step 2: Stream ingestion`
  $D_{clean} \leftarrow$ Preprocess(D_t) ; `// Step 2: Cleaning & anonymization`
  StoreVersioned(D_{clean}) ; `// Step 2: DVC + MinIO snapshot`
  (F_t, replacementFlag) $\leftarrow$ SHAPFeatureAdapter(F_{t-1}, D_{clean}, τ_{shap}) ; `// Step 3: Feature adaptation`
  drift $\leftarrow$ MonitorDrift(D_{clean}, D_{ref}, τ_{PSI}) ; `// Step 7: PSI-based drift check`
  degraded $\leftarrow$ CheckPerformance($\mathrm{F1}_{threshold}$) ; `// Step 8: SLA compliance`
  unfair $\leftarrow$ AuditFairness($\Delta\mathrm{DP}_{threshold}$) ; `// Step 9: Bias auditing`
  feedback $\leftarrow$ PollFeedbackEvents() ; `// Step 10: Human-in-the-loop check`
  if *replacementFlag* ***or*** *drift* $> \tau_{PSI}$ ***or*** *degraded* ***or*** *unfair* ***or*** *feedback* **then**
    $M_t \leftarrow$ Retrain(D_{clean}, F_t) ; `// Step 4: Model retraining`
    metrics $\leftarrow$ Evaluate(M_t) ; `// Step 4: Evaluation`
    if *metrics.pass_sla* **then**
      DeployModel(M_t) ; `// Step 12: CI/CD deployment`
      $D_{ref} \leftarrow$ UpdateReference(D_{clean}) ; `// Step 7: Drift baseline update`
  LogTelemetry() ; `// Step 7: Metric logging`
  GenerateComplianceReport() ; `// Step 9: Audit documentation`
  NotifyStakeholders() ; `// Step 13: Slack/Trello alerts`

Algorithm 2: Algorithm S2: SHAP_Feature_Adapter

Input: Feature set F, Data batch D, Threshold τ_{shap}

Output: Updated feature set F', Replacement flag

```
F' ← F ;                                      // Initialize updated feature set
replacement_flag ← False ;                    // No changes initially
health ← CheckFeatureHealth(F) ;              // Status from external API
for f ∈ F do
    φ_current ← ComputeSHAP(f, D) ;           // SHAP attribution
    φ_hist ← LoadSHAPHistory(f) ;             // 30-day SHAP values
    Δφ ← Mean(|φ_current − φ_hist|) ;         // Mean SHAP deviation
    volatility ← Std(φ_hist) ;                // Historical variance
    if health[f] == DEPRECATED or Δφ > τ_shap or volatility
      > τ_shap then
        candidates ← QueryFeatureStore(f) ;   // Semantic match
          by metadata
        ranked ← [ ] ;                        // Ranked list init
        for c ∈ candidates do
            φ_c ← ComputeSHAP(c, D);
            sim ← CosineSimilarity(φ_current, φ_c);
            Append(ranked, (c, sim));
        Sort(ranked) by sim descending;
        if ranked[0].sim > 0.85 then
            top ← ranked[0].c;
            F' ← (F' \ {f}) ∪ {top};
            replacement_flag ← True;
            LogReplacement(f, top, sim);
            if sim < 0.93 then
                TriggerHumanReview(f, top);
return F', replacement_flag;
```

Supplementary Note 3: Pipeline Step Complexity Details

Step	Complexity Analysis
**1**	$T_{\text{registry}} = \mathcal{O}(m \cdot \log v)$. Complexity grows logarithmically with version history, ensuring scalability.
**2**	$T_{\text{preprocess}} = \mathcal{O}(n \cdot k)$.where n Preprocessing grows linearly with records and operations, suitable for Spark parallelization.
**3**	$T_{\text{feature}} = \mathcal{O}(m \cdot f)$.Feature engineering scales linearly, but SHAP adds attribution cost.
**4**	$T_{\text{train}} = \mathcal{O}(n \cdot f \cdot e)$. Training complexity rises with data size and feature count; distributed GPUs mitigate bottlenecks.
**5**	$T_{\text{registry}} = \mathcal{O}(m \cdot \log v)$. Logarithmic complexity with version history.
**6**	$T_{\text{serve}} = \mathcal{O}(f + i + r)$. Inference latency dominated by model forward pass; optimized under 50 ms in experiments.
**7**	$T_{\text{drift}} = \mathcal{O}(n \cdot m)$. Complexity grows with monitored features but is parallelizable.
**8**	$T_{\text{alert}} = \mathcal{O}(p \cdot t)$. Alert checking scales with metrics and evaluation frequency.
**9**	$T_{\text{audit}} = \mathcal{O}(m \cdot d \cdot k)$. Complexity grows with groups/metrics; audits feasible under batch evaluation.
**10**	$T_{\text{feedback}} = O(u \cdot r)$. Scales linearly with feedback submissions and response types.
**11**	$O(1)$. Constant time for trigger evaluation (simple condition checks).
**12**	$T_{\text{deploy}} = O(m \cdot v + d)$. Scales with microservices and version checks plus delivery time.
**13**	$T_{\text{notify}} = O(s \cdot n)$. Scales with stakeholder roles and event types.
